# 3D Printing of Shape Memory Resin for Orthodontic Aligners with Green Synthesized Antimicrobial ZnO Nanoparticles Coatings: Toward Bioactive Devices

**DOI:** 10.3390/bioengineering12111193

**Published:** 2025-11-01

**Authors:** Airy Teramoto-lida, Rafael Álvarez-Chimal, Lorena Reyes-Carmona, Marco Antonio Álvarez-Pérez, Amaury Pozos-Guillen, Febe Carolina Vázquez-Vázquez

**Affiliations:** 1 Orthodontics Department, División de Estudios de Posgrado e Investigación, Facultad de Odontología, Universidad Nacional Autónoma de México, Mexico City 04510, Mexico; airyteramoto@fo.odonto.unam.mx; 2 Laboratorio de Bioingeniería de Tejidos, División de Estudios de Posgrado e Investigación, Facultad de Odontología, Universidad Nacional Autónoma de México, Ciudad Universitaria, Coyoacán, Mexico City 04510, Mexico; malva@fo.odonto.unam.mx; 3 Laboratorio de Investigación en Materiales Dentales y Biomateriales, División de Estudios de Posgrado e Investigación, Facultad de Odontología, Universidad Nacional Autónoma de México, Ciudad Universitaria, Coyoacán, Mexico City 04510, Mexico; rachimal65@comunidad.unam.mx; 4 Laboratorio de Biointerfases, División de Estudios de Posgrado e Investigación, Facultad de Odontología, Universidad Nacional Autónoma de México, Ciudad Universitaria, Coyoacán, Mexico City 04510, Mexico; lorena.unam753@gmail.com; 5 Laboratorio de Ciencias Básicas, Facultad de Estomatología, Universidad Autónoma de San Luis Potosí, Av. Dr. Manuel Nava No. 2, San Luis Potosí 78290, Mexico; apozos@uaslp.mx

**Keywords:** ZnO nanoparticles, 3D printing, dental resin, green synthesis, biocompatibility, antibacterial

## Abstract

The development of bioactive dental materials with antimicrobial and biocompatible properties is important for improving clinical outcomes and reducing complications associated with intraoral devices. This study presents a novel approach that combines a 3D-printed shape-memory resin (TC-85DAC) with green-synthesized zinc oxide nanoparticles (ZnO NPs) to enhance biological performance. ZnO NPs were synthesized using *Dysphania ambrosioides* extract, producing quasi-spherical particles with a crystalline hexagonal structure and sizes between 15 and 40 nm. Resin discs were coated with ZnO NPs at 10%, 20%, and 30%, then assessed for biocompatibility with human gingival fibroblasts and antibacterial activity against *Porphyromonas gingivalis* and *Streptococcus mutans*. Surface roughness was also considered with and without ZnO NPs. Biocompatibility assays revealed a concentration- and time-dependent increase in cell viability, with the highest values at 30% ZnO NPs after 72 h of exposure to the NPs. Antibacterial testing confirmed the inhibition of both species, with *Porphyromonas gingivalis* showing greater sensitivity. Surface roughness increased with higher ZnO NPs concentrations, significantly influencing biological interactions. The integration of green-synthesized ZnO NPs with shape-memory resin produced a multifunctional dental material with improved bioactivity. This sustainable strategy enables bioactive coatings on 3D-printed resins, with potential applications in the next generation of smart dental devices.

## 1. Introduction

Orthodontic aligners are a treatment modality for dental malocclusions, offering advantages over conventional fixed appliances, including improved esthetics and patient comfort [1,2]. Despite their widespread adoption, challenges such as bacterial biofilm accumulation and suboptimal fit limit their long-term efficacy and patient compliance [3]. Moreover, the additive manufacturing process inadvertently introduces microscopic spaces that could serve as potential niches for bacteria, further exacerbating the issue of oral hygiene maintenance [4].

Bacterial colonization on aligner surfaces not only contributes to enamel demineralization and periodontal inflammation but also compromises device performance due to material degradation [5]. In particular, bacterial species such as *Streptococcus mutans*, a relevant pathogen in dental caries, and *Porphyromonas gingivalis*, a major pathogen in periodontal infections, have been identified on orthodontic materials, highlighting their potential role in disrupting the oral microbiota and compromising oral health during orthodontic treatment. Although clear aligner therapy has been described as better than conventional fixed appliances for maintaining periodontal health, grooves, ridges, microcracks, and surface abrasions present on aligner materials may provide an ideal environment for bacterial adhesion and plaque biofilm formation [6,7]. Consequently, there is a need for innovative solutions that enhance the antimicrobial properties while maintaining the biocompatibility and functionality of orthodontic aligners.

Recent advancements in additive manufacturing and smart materials have provided new opportunities for developing next-generation dental devices with tailored properties [8]. Among these, 3D-printed shape memory polymers have gained attention for their ability to undergo programmable deformation and recover their original configuration upon external stimuli, such as temperature or light [9,10]. When applied to orthodontic aligners, shape memory polymers can improve force delivery and fit accuracy by adapting to dynamic tooth movements, thereby optimizing treatment efficiency [11]. TC-85DAC resin is a biocompatible light-curing polyester-urethane polymer used for the direct printing of aligners [12]. One of its properties is shape memory, which allows it to deform and return to its original shape at oral cavity temperature [13]. However, the integration of bioactive functionalities, such as antimicrobial activity, into shape-memory polymer-based aligners remains less explored.

The light-cured polyetherurethane polymer as the TC-85DAC resin has emerged as an advanced acrylic-based material in dentistry, offering excellent flexibility, durability, and shape-memory properties, making it suitable for 3D-printed orthodontic aligners and other bioactive applications, as was mentioned before. Alongside this system, acrylic binders, such as aryloxy phosphazene [14] or carboxy phosphazene methacrylates [15], are frequently employed in dental materials due to their film-forming ability, transparency, and chemical versatility, which have shown enhanced mechanical strength, thermal stability, and biocompatibility [16]. However, regardless of polymer type, these materials inherently lack antimicrobial properties.

Nanomaterials, such as nanoparticles, have revolutionized biomedical applications due to their unique physicochemical properties, including a high surface-to-volume ratio, tunable surface chemistry, and enhanced mechanical and biological interactions [17]. In dentistry, nanoparticles have been extensively studied for their ability to provide antibacterial, remineralizing, and drug-delivery capabilities in polymeric matrices [18,19]. Their incorporation can enhance the mechanical strength, surface properties, wear resistance, and bioactivity while minimizing adverse biological effects [20,21].

Among these nanomaterials, zinc oxide nanoparticles (ZnO NPs) have demonstrated broad-spectrum antimicrobial activity against common oral pathogens while maintaining biocompatibility [22,23]. The incorporation of these compounds into dental materials is promising for reducing biofilm formation and preventing caries [24]. When applied as a coating or embedded within 3D-printed shape memory polymer aligners, ZnO NPs can provide sustained antimicrobial protection without compromising the orthodontic performance [25].

ZnO NPs have antimicrobial efficacy, biocompatibility, and chemical stability, which make them particularly suitable for biomedical and dental applications [24]. Compared with metallic nanoparticles, such as Ag or Cu, ZnO NPs exhibit broad-spectrum antibacterial activity through ion release and reactive oxygen species generation, while exhibiting lower cytotoxicity to human cells compared to metallic nanoparticles [26]. Unlike polymer-based nanoparticles, which primarily act as passive carriers [27], ZnO NPs possess intrinsic bioactivity, providing sustained antimicrobial protection without compromising the mechanical or optical properties of the resin. Furthermore, compared to natural antimicrobial compounds, such as chitosan [28] or essential oils [29], ZnO NPs offer superior durability, controlled release behaviour, and resistance to degradation under oral conditions [24].

This study aimed to demonstrate the synergistic combination of a 3D-printed shape-memory resin and ZnO NPs coatings to develop bioactive orthodontic aligners with enhanced antimicrobial properties. ZnO NPs were green-synthesized, which is a novel and eco-friendly technique [17]. The size and shape of the nanoparticles were confirmed and used to coat the 3D-printed memory resin models. Biocompatibility and antibacterial efficacy were evaluated to fabricate bioactive devices, considering the roughness of the surface generated by the ZnO NPs, establishing a proof of concept for clinically viable multifunctional aligners that offer a new generation of smart orthodontic devices to improve the treatment outcomes.

## 2. Materials and Methods

### 2.1. Materials

Dried *Dysphania ambrosioides* leaves were commercially obtained and used for the green synthesis of ZnO NPs. Zinc nitrate hexahydrate (Zn(NO_3_)_2_·6H_2_O, ≥99% purity) was purchased from Sigma-Aldrich (St. Louis, MO, USA). TC-85DAC resin (Graphy, Seoul, Republic of Korea) was used to fabricate the discs used in this study. For biocompatibility evaluation, Dulbecco’s Modified Eagle’s Medium (DMEM), fetal bovine serum (FBS), penicillin, streptomycin, fungizone, and WST-1 assay kit were purchased from GIBCO (Thermo Fisher Scientific, Waltham, MA, USA). Trypticase soy agar (TSA; BD Bioxon, Mexico City, Mexico) and defibrinated sheep blood were used for antibacterial testing.

### 2.2. Synthesis and Characterization of ZnO NPs

ZnO NPs were green synthesized using the following method [30]: 20 g of dried *Dysphania ambrosioides* leaves were soaked in 200 mL of deionized water for 2.5 h at room temperature and then stirred for 2 h at 40 °C and 50 rpm.

**Figure 1:**
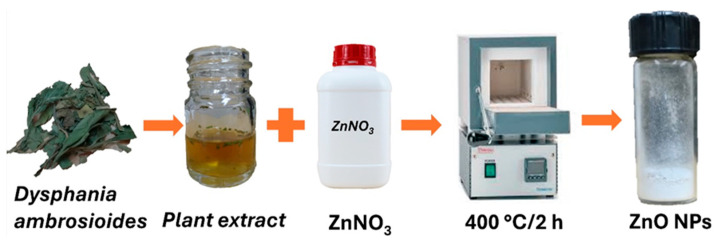
Green synthesis of the ZnO NPs.

For the synthesis process, 100 mL of the previously obtained extract was mixed with 3.5 g of Zn(NO_3_)_2_ · 6H_2_O and stirred at 60 °C for 2 h at 1500 rpm. The resulting precipitate was separated by centrifugation at 8000 rpm for 10 min, washed thrice with deionized water, and dried overnight in a hot-air oven at 50 °C. After drying, the precipitated powder was annealed at 400 °C for 2 h to produce a pale white powder, which were the ZnO NPs (Figure 1).

The powder produced after the synthesis was analyzed using X-ray diffraction (XRD), field-emission scanning electron microscopy (FESEM), and transmission electron microscopy (TEM).

XRD was employed to confirm the synthesis of ZnO and investigate its crystalline structure using a Bruker (Massachusetts, USA) AXS D8 Advance X-ray diffractometer operating at 40 kV and 30 mA, utilizing Cu K radiation (1.541) across 2θ angles ranging from 4° to 110°. The XRD data were indexed using the powder diffraction file (PDF) #891397 for ZnO. The average crystallite size was calculated using the Debye–Scherrer equation:

D = K λ/Bcosθ

where D: average crystalline size; K: shape factor usually taken as 0.9; λ: wavelength of the X-ray radiation (k = 1.541 Å) for λKαCu; θ: Bragg diffraction angle; Β: line width at a half-maximum height.

For FESEM, the samples were placed on an aluminum holder with conductive carbon tape. A JEOL (Tokyo, Japan) JSM7800F FESEM model with a resolution of 0.7 nm was used.

For TEM analysis, the sample was placed on a 300-mesh carbon-coated copper grid. A JEOL transmission electron microscope (JEM 2010 FEG with 0.19 nm resolution). The interplanar distance measurements were obtained from the TEM images using the Fast Fourier Transform (FFT) in the Digital Micrograph 3 software from GATAN.

### 2.3. Design and 3D Printing of the Resin Disc for Biocompatibility and Antibacterial Evaluations

The discs were designed using TinkerCAD software (Autodesk Inc., San Francisco, CA, USA) with dimensions of 10 × 10 × 1 mm. Seven supports were added to each model using the CHITUBOX software (version 1.9.1, Shenzhen, Guangdong, China). The fabrication was performed using TC-85DAC resin (Graphy, Seoul, Republic of Korea, viscosity of 500 ± 100 cps at 25 °C, 405 nm wavelength for photocuring) and a Uniz NBEE LED 3D printer (3D Uniz 4K, Uniz, San Diego, CA, USA), operating at a wavelength of 385–405 nm, in accordance with the manufacturer’s instructions.

Post-processing involved detaching the disc from the build platform and drying them with compressed air to remove excess uncured resin. The support structures were manually removed. The specimens were cured for 5 min in a nitrogen-generating curing unit (Tera Harz Cure THC2; Graphy, Seoul, Republic of Korea). After curing, the samples were cleaned in an ultrasonic bath at 80 °C for 2 min, followed by immersion in boiling water for 1 min. Finally, the discs were air-dried at room temperature for 15 min.

### 2.4. Coating the 3D-Printed Resin Disc with ZnO NPs

Suspensions of the synthesized ZnO NPs were prepared at concentrations of 10%, 20%, and 30%, as described previously [31,32]. Using the suspension dispersion coating method, 10 µL of each suspension was applied to the surface of the 3D-printed resin discs. The coated discs were photocured according to the manufacturer’s recommended protocol, which enhanced their mechanical strength and surface hardness.

The presence and distribution of ZnO NPs on the disc surfaces were visually confirmed using an optical microscope (Olympus CX43, Olympus Corp., Tokyo, Japan) at 40× magnification.

### 2.5. Surface Roughness Test

Surface roughness was evaluated using a portable Mitutoyo Surftest SJ-210 profilometer (Mitutoyo, Kawasaki, Japan) calibrated according to the manufacturer’s specifications. Measurements were performed in triplicate for each sample (3D-printed resin discs coated and uncoated with ZnO NPs). The device was set up using a diamond stylus with a tip radius of 2.5 µm, contact force of 5 N, and cut-off of 0.8 mm, in accordance with the ISO 21920 (Geneva, Switzerland) guidelines.

### 2.6. Biocompatibility Evaluation

#### 2.6.1. Cell Culture

Human gingival fibroblasts (hGFs; ATCC PCS-201-018) were used for the biocompatibility assay. Cells were cultured in Dulbecco’s Modified Eagle Medium (DMEM) supplemented with 10% fetal bovine serum (FBS), penicillin (1000 U/mL), streptomycin (100 µg/mL), and fungizone (0.3 µg/mL). Cultures were maintained at 37 °C in a humidified incubator with 5% CO_2_ and 95% air. Cells were used for experiments after reaching confluence, typically within 5–7 days [33].

#### 2.6.2. WST-1 Assay

The biocompatibility of the 3D-printed resin discs coated with ZnO NPs was evaluated using the WST-1 assay [33]. hGFs were seeded at a density of 1 × 10^4^ cells/mL onto the coated discs in triplicate and incubated for 48 and 72 h. The WST-1 assay measures cell viability based on the activity of mitochondrial succinate-tetrazolium reductase, which reduces the WST-1 salt to a formazan dye. The amount of formazan is directly proportional to the number of metabolically active cells.

After the incubation period, 400 µL of fresh culture medium and 40 µL of WST-1 reagent were added to each well, followed by incubation for 4 h at 37 °C. Subsequently, 200 μL of the supernatant was transferred to a new well, and the absorbance was measured at 545 nm using a microplate reader (ChroMate; Awareness Technology, Palm City, FL, USA).

### 2.7. Antibacterial Evaluation

The antibacterial activity of the ZnO NPs-coated 3D-printed resin discs was assessed using the disc diffusion method (Kirby–Bauer assay) [34,35,36]. Discs coated with ZnO na-noparticles at concentrations of 10%, 20%, and 30% were tested against two oral patho-genic bacterial strains: *Streptococcus mutans* (Gram-positive ATCC 25275) and *Porphy-romonas gingivalis* (Gram-negative ATCC 33277).

Bacterial suspensions were standardized to a concentration of 1 × 10^9^ cells/mL and cultured on tryptic soy agar (TSA) supplemented with 5% defibrinated sheep blood. The ZnO-coated discs were placed on inoculated agar plates in triplicate. Chlorhexidine (0.2%) served as the positive control, and uncoated 3D-printed resin discs (without ZnO NPs) served as the negative control. The plates were incubated under anaerobic conditions (a gas mixture of N_2_ and CO_2_) for 7 days. Following incubation, the zones of bacterial inhibi-tion surrounding the discs were measured in millimetres to evaluate the antibacterial ef-ficacy.

### 2.8. Statistical Analysis

Data are presented as mean ± standard deviation. GraphPad 8 statistical software was used to analyze the data with ANOVA tests to find statistically significant differences between the groups evaluated, with *p* ≤ 0.05.

## 3. Results

### 3.1. Green Synthesis and Characterization of ZnO NPs

**Figure 2:**
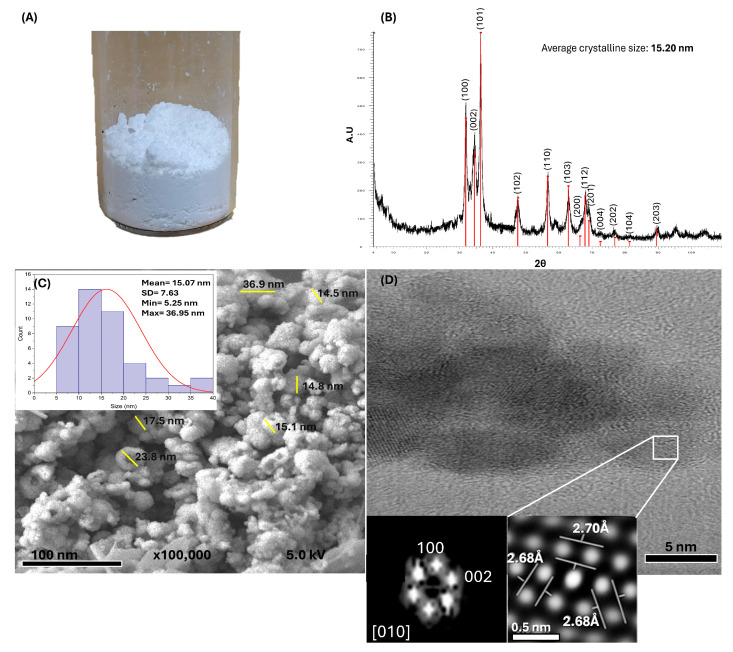
Characterization of green-synthesized ZnO NPs: (**A**) Image of the ZnO NPs obtained via green synthesis showing a white powder. (**B**) X-ray diffraction pattern of the ZnO NPs, confirming their hexagonal wurtzite crystalline structure. The average crystallite size was calculated to be 15.20 nm using the Debye–Scherrer equation. (**C**) Field-emission scanning electron microscopy image showing quasi-spherical ZnO NPs with sizes ranging from 5 to 36 nm (yellow lines), forming visible aggregates.; the size distribution analysis is inserted in the figure. (**D**) Transmission electron microscopy image of the ZnO NPs. The inset shows the crystallographic analysis (crystalline planes and interplanar distances), further confirming the hexagonal crystalline structure.

The white powder obtained from the green synthesis (Figure 2A) was analyzed using multiple analytical techniques, which confirmed the successful formation of ZnO NPs. XRD analysis (Figure 2B) revealed characteristic peaks corresponding to the reflections of the hexagonal wurtzite crystal pattern, which is consistent with the reference standard PDF #89-1397. The sharp and well-defined diffraction peaks indicate high crystallinity and the absence of secondary phases or impurities. The average crystal size, calculated using the Debye-Scherrer equation, was approximately 15.20 nm, demonstrating that the synthesis method effectively controlled the nanoscale crystal growth.

FESEM images (Figure 2C) showed quasi-spherical nanoparticles with sizes ranging from 5.25 to 36.95 nm, with an average size of 15.07 ± 7.63 nm confirming nanoscale morphology. TEM analysis (Figure 2D) further corroborated these observations, providing detailed insights into the particle size and shape and revealing the hexagonal crystalline structure of ZnO, which is consistent with the XRD results.

### 3.2. Design, 3D Printing of the Resin Disc, and Coatings with ZnO NPs

**Figure 3:**
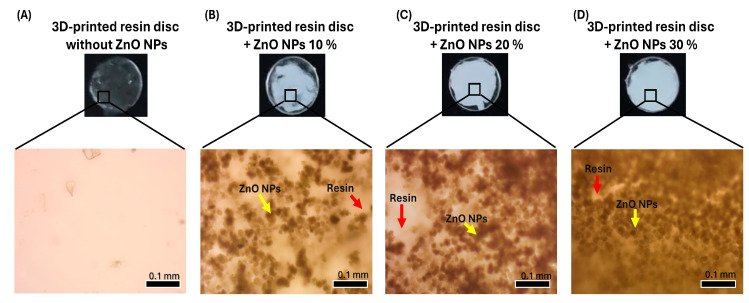
Surface appearance and microscopic evaluation of 3D-printed resin discs coated with varying concentrations of ZnO NPs. (**A**) Uncoated resin disc showing a clean and transparent surface, as confirmed by optical microscopy. (**B**) Resin disc coated with 10% ZnO NPs, displaying partial surface coverage with visible white areas; microscopic analysis revealed the presence of ZnO NP aggregates (yellow arrow), the clear areas correspond to the resin (red arrow). (**C**) Resin disc coated with 20% ZnO NPs, with most of the surface covered in white; optical microscopy shows increased aggregation of ZnO NPs (yellow arrow); the clear areas correspond to the resin (red arrow). (**D**) Resin disc coated with 30% ZnO NPs, exhibiting complete surface coverage; microscopy reveals a higher density of ZnO NP aggregates (yellow arrow), and the remaining clear areas correspond to the resin (red arrow).

Discs measuring 10 × 10 × 1 mm were 3D printed because of the versatility of the model and the ease of surface coating with the ZnO NPs. Once the discs and ZnO NP suspensions at different concentrations were prepared, the nanoparticles were applied to the disc surfaces via a uniform dispersion method (Figure 3A–D). The formation of a white layer and the distribution of ZnO NPs were confirmed both visually and by optical microscopy. As the concentration increased, greater surface coverage and more pronounced nanoparticle aggregation were observed.

### 3.3. Surface Roughness Test

The surface roughness (Ra) values obtained for each experimental group are listed in Table 1. An increase in roughness was observed as the concentration of ZnO NPs increased. The group of resin discs coated with 30% ZnO NPs exhibited higher roughness than the other groups.

### 3.4. Biocompatibility Evaluation

**Figure 4:**
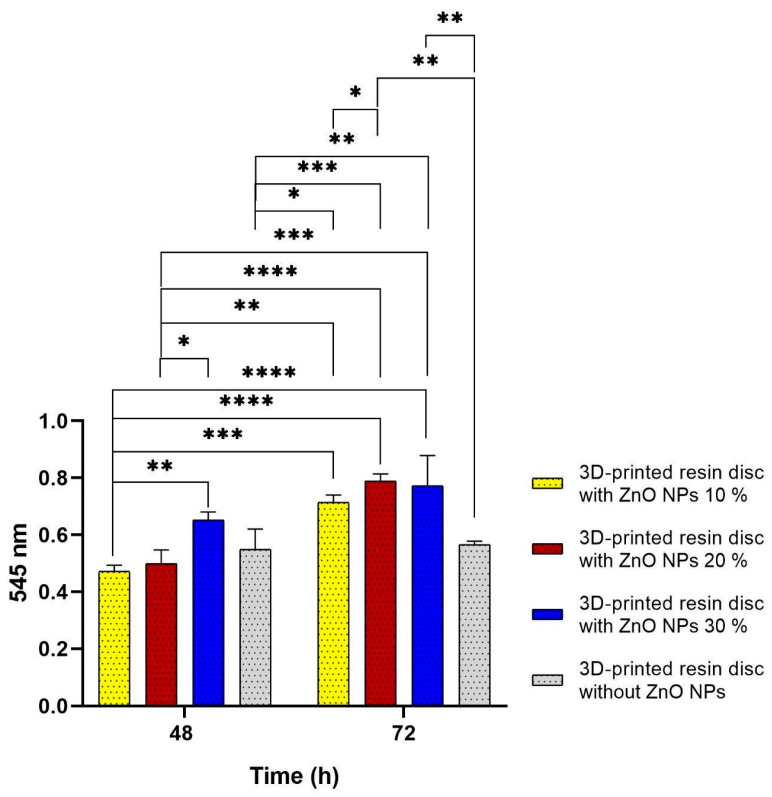
Biocompatibility evaluation of 3D-printed resin discs coated with ZnO NPs at 10% (yellow), 20% (red), and 30% (blue) concentrations, and control (orange) after 48 and 72 h of exposure to hGFs. Statistically significant differences were observed at 72 h, particularly in the 20% and 30% ZnO NPs groups compared to earlier time points and other concentrations (* *p* < 0.05, ** *p* < 0.01, *** *p* < 0.001, **** *p* < 0.0001).

The biocompatibility results (Figure 4) demonstrated a concentration-dependent response to ZnO NPs coatings on 3D-printed resin discs after 48 and 72 h. At 48 h, discs coated with 10% ZnO NPs maintained high cell viability, indicating that lower concentrations did not induce noticeable cytotoxicity and were well tolerated by the cells. The 20% ZnO NPs group showed even higher viability than the 10% group, and the 30% ZnO NPs coating exhibited the highest biocompatibility, with statistically significant differences compared to the 20% group.

These findings contrast with those of various reports, where higher concentrations of ZnO NPs were associated with reduced cell viability due to reactive oxygen species (ROS) generation [37,38,39]. In this study, however, the opposite trend was observed, suggesting that at concentrations between 20% and 30%, ZnO NPs may promote cellular proliferation or benign adaptation, which could be attributed to the controlled release of Zn^2+^ ions, which are known to support cellular repair and regeneration processes [40,41].

The control group (uncoated resin discs) also exhibited high cell viability, confirming the biocompatibility and suitability of the base resin for dental applications. A slight decrease in cell viability was observed in the 10% and 20% ZnO NPs groups at 48 h compared to the control, although these differences were not statistically significant. This may reflect a transient adaptation response to the modified surface, without cytotoxic effects [42,43].

By 72 h, all ZnO NPs-coated groups showed improved cell viability, with statistically significant increases compared to the 48 h values. This trend suggests a delayed cellular response, potentially due to the stabilization of the cell–material interface and modulation of the local microenvironment [36]. In contrast, the control group exhibited no significant change in viability between 48 and 72 h, indicating a more stable but less responsive profile.

Notably, the 20% and 30% ZnO NPs-coated discs reached similar levels of biocompatibility at 72 h. These results suggest that this concentration range may represent an optimal balance for enhancing cell–material interactions without compromising cell health. Overall, this supports the potential of ZnO NPs as bioactive surface modifiers for 3D-printed dental materials, promoting compatibility with oral tissues and improving their biological performance [44].

### 3.5. Antibacterial Evaluation

The antimicrobial activity of 3D-printed resin discs coated with ZnO NPs was evaluated against two oral pathogens: *Porphyromonas gingivalis* and *Streptococcus mutans.* The results demonstrated significant antibacterial effects for all tested ZnO NPs concentrations.

**Figure 5:**
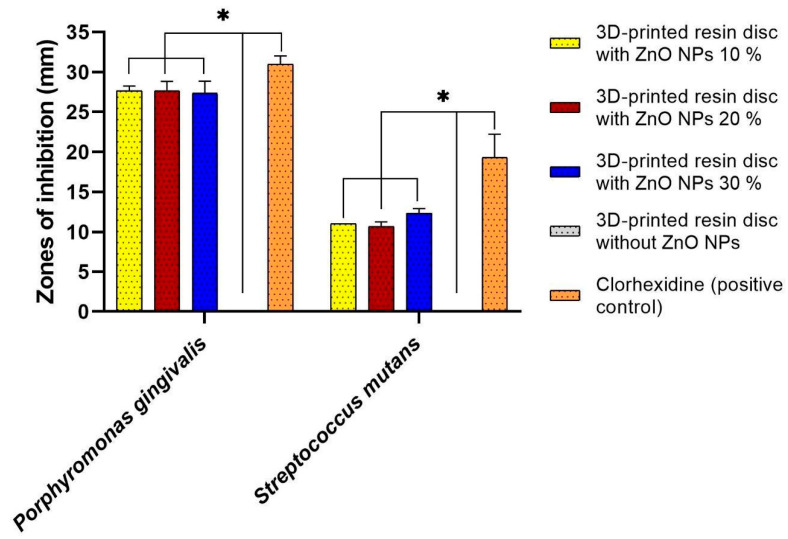
Antibacterial activity of 3D-printed resin discs coated with ZnO NPs at 10% (yellow), 20% (red), and 30% (blue) concentrations, and control (orange, not observed) against *Porphyromonas gingivalis* and *Streptococcus mutans*. Inhibition zones were measured using the agar diffusion method after 7 days. *Porphyromonas gingivalis* exhibited significantly larger zones of inhibition (>25 mm) compared to *Streptococcus mutans* (>10 mm) across all concentrations. The results are expressed as mean ± standard deviation (* *p* < 0.0001).

**Figure 6:**
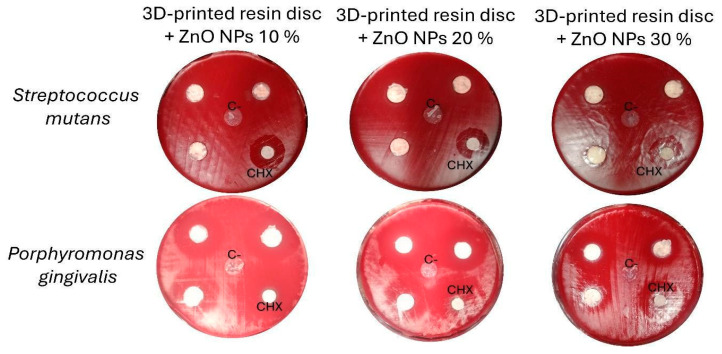
Inhibition zones produced by 3D-printed resin discs coated with ZnO NPs at concentrations of 10%, 20%, and 30% against *Porphyromonas gingivalis* and *Streptococcus mutans*. C–: negative control (uncoated 3D-printed resin disc); CHX: positive control (chlorhexidine).

Notably, *Porphyromonas gingivalis* exhibited high sensitivity, with inhibition zones exceeding 25 mm, whereas *Streptococcus mutans* showed smaller inhibition zones, consistently greater than 10 mm (Figure 5 and Figure 6). The observed inhibition patterns were consistent across all concentrations (10%, 20%, and 30%), indicating that within this range, the antibacterial activity of the ZnO NPs coatings was effective but not concentration-dependent. These findings suggest that ZnO NPs provide broad-spectrum antibacterial properties when incorporated into 3D-printed dental resins, particularly effective against Gram-negative bacteria such as *Porphyromonas gingivalis*.

## 4. Discussion

This study evaluated the effectiveness of incorporating ZnO NPs into resin for orthodontic aligners.

The green synthesis approach employed here offers advantages over conventional physicochemical methods such as chemical precipitation [45], solvothermal synthesis [46], and microwave-assisted methods [47], which typically require high temperatures [48], toxic reagents [49], or strictly controlled conditions [38]. In contrast, green synthesis uses biological extracts as reducing and stabilizing agents, thereby minimizing hazardous substances, reducing toxic waste generation, and yielding products with lower environmental impact and enhanced biocompatibility [17,50,51].

Although various studies have reported the synthesis of ZnO NPs using traditional methods [52,53], these methods often involve synthetic surfactants or complex purification steps [53]. Nanoparticles synthesized through green routes are frequently coated with organic compounds derived from plant extracts [54], enhancing their colloidal stability and surface functionality. These characteristics facilitate their application in the dental, biomedical, pharmaceutical, and environmental fields without the need for further modifications [55]. Green synthesis using plant species such as *Medicago sativa* (yielding quasi-spherical nanoparticles ~10 nm) [56], *Limonia acidissima* (12–45 nm) [57], *Solanum torvum* (30–40 nm) [58], *Ipomoea aquatica* (50–200 nm) [59] has demonstrated the use of a green approach allows for ZnO NPs synthesis with controlled sizes and defined shapes using a simpler, more economical, and sustainable methodology, practically in a single step [60,61].

*Dysphania ambrosioides* is a popular medicinal plant in many countries in Latin America and the Caribbean. It is used as a remedy for bronchitis, healing wounds, and stomach aches. Previous studies have demonstrated that the organic compounds in *Dysphania ambrosioides* participate in the synthesis of ZnO-NPs. The oxidation of OH to carboxyl groups is involved in the stabilization of nanoparticles, in addition to controlling their shapes [35]. Proteins, organic acids, vitamins, flavonoids, alkaloids, polyphenols, terpenoids, heterocyclic compounds and polysaccharides also contribute to the reduction of metal salts, in addition to surrounding and stabilizing the synthesized ZnO NPs [30].

Prior studies have demonstrated that the inclusion of nanoparticles significantly improves the bacterial biofilm resistance of thermoformed aligners [62,63] and 3D printed clear aligners [4]. In this study, Graphy resin TC-85DAC was selected for functionalization due to its favourable physical properties, particularly its shape memory at oral temperature, which improves adaptation, reduces force decay, and enables greater tooth movement per step. Its microhardness is similar to that of thermoformed sheets, ensuring the durability and effectiveness of dental aligners [13]. Importantly, the mechanical stability of 3D printed Shape Memory Aligners remains after one week of intraoral use [64], potentially minimizing gaps between the resin and tooth surface where bacterial colonization can occur. As a printed shape memory polymer [65], TC-85DAC enables innovative applications, such as self-adjusting aligners that can respond to thermal changes in the oral cavity [66,67]. However, despite these advantages, TC-85DAC lacks inherent antimicrobial properties.

The findings demonstrate that incorporating ZnO NPs into coatings for 3D-printed dental resins does not compromise biocompatibility and, depending on the concentration and exposure duration, may even enhance cellular interaction. These outcomes are consistent with previous studies that reported improved biocompatibility and other functional properties following the addition of nanoparticles, such as zirconia [68,69] or ZnO [70,71]. At higher concentrations, ZnO NPs can release Zn^2+^ ions; this controlled ionic environment supports cellular metabolic activity, rather than toxicity [72]. The increased ZnO NPs content modified the surface morphology, which enhanced fibroblast adhesion [73]. Cells sense these topographical cues through focal adhesions, which activate intracellular pathways linked to cytoskeletal organization and proliferation [74] ZnO NPs also increases the hydrophilicity of the polymer surface and improves protein adsorption (such as fibronectin or vitronectin) that facilitates fibroblast attachment and spreading, which are essential for promoting cell proliferation, migration, and protein synthesis in fibroblasts [75].

Concentrations of 10%, 20%, and 30% were selected based on prior research, where these concentrations were found to be effective in conferring antimicrobial properties to the coated materials [31,32,76]. Even at lower concentrations, significant antibacterial activity was observed, indicating a potential saturation threshold beyond which no further increase in efficacy occurs [77].

A key observation was that *Porphyromonas gingivalis* exhibited greater sensitivity to ZnO NPs than did *Streptococcus mutans*. The antimicrobial effect against *Porphyromonas gingivalis* was comparable to that of chlorhexidine, a widely used dental antiseptic [78]. Although statistically significant differences were observed between the groups. This sensitivity is likely due to the structural composition of *Porphyromonas gingivalis*, a Gram-negative bacterium with a thinner peptidoglycan layer and an outer membrane that is more susceptible to oxidative stress induced by reactive oxygen species (ROS) generated by ZnO NPs. Additional mechanisms, such as Zn^2+^ ion release, membrane lipid peroxidation, and nanoparticle surface accumulation, contribute to bacterial membrane disruption and cell death [79,80].

In contrast, *Streptococcus mutans*, a Gram-positive bacterium, exhibited reduced sensitivity. Its thick peptidoglycan cell wall likely acts as a protective barrier against nanoparticle penetration and ROS damage [81]. This observation aligns with earlier reports indicating that Gram-positive organisms generally exhibit greater resistance to nanoparticles than Gram-negative species [82,83,84].

Uncoated 3D-printed resin discs exhibited no antibacterial activity, confirming the absence of inherent antimicrobial properties in the base resin. Functionalization with ZnO NPs introduces bioactive functionality, enabling the material to inhibit pathogenic bacteria associated with dental caries and periodontal diseases. This advancement addresses a significant limitation of printed dental resins, which, while widely used in direct restorations, provisional devices, and custom-fitted prostheses, are often prone to biofilm accumulation and secondary infections [85].

Previous studies have investigated nanoparticle-coated aligners and demonstrated their significant antimicrobial properties [62,63]. Traditional coating methods, such as cathodic sputtering, face challenges, including high equipment costs, difficulty in controlling stoichiometry, and limited temperature control [86,87]. In contrast, the suspension dispersion coating technique used in this study is simple, cost-effective, and yields coatings with uniform distribution [88,89].

The surface roughness generated by the ZnO NPs coatings increased with increasing concentration of ZnO NPs, significantly influencing the biological interactions of the material. Increased micro- and nanoscale roughness enhances fibroblast adhesion and proliferation by providing a more favourable topographical environment for cell anchorage and spreading [73]. At the same time, these irregularities promote closer contact between bacteria and nanoparticles, strengthening the antimicrobial effect through Zn^2+^ ion release and reactive oxygen species generation, thereby hindering biofilm formation and reducing bacterial viability without compromising biocompatibility [72].

As the ZnO NP concentration increased, the probability of particle–particle interactions (rather than particle–matrix interactions) increased. This leads to agglomeration; clusters of ZnO NPs form instead of remaining evenly dispersed. These clusters create microscopic peaks and valleys, increasing surface roughness; when more ZnO NPs are incorporated, surface heterogeneity increases; some regions are ZnO-rich, while others remain polymer-rich [90]. Increasing the ZnO NP concentration enhances the surface roughness mainly owing to particle aggregation, uneven dispersion, and local stress gradients during curing or drying [91]. These physical effects dominate the expected balancing effect of the bioactive system, leading to rougher but more bioactive surfaces.

Although aligner aesthetics are a key consideration for patients, the white coating layer formed by ZnO NPs may compromise their esthetic appeal. Future investigations are needed to determine whether selective coating, for example, on occlusal or lingual surfaces, can maintain antimicrobial efficacy while preserving visual appearance.

These results also underscore the dual benefits of ZnO NPs incorporation: antimicrobial activity and high biocompatibility. These findings support the development of ZnO-functionalized 3D-printed resins as next-generation dental materials that not only restore mechanical function but also promote oral health through active biointervention.

## 5. Conclusions

This study presents the development of a novel bioactive system based on 3D-printed shape-memory resin (TC-85DAC) discs coated with ZnO NPs synthesized using a green approach. The incorporation of ZnO NPs significantly enhanced the biological performance of the resin and imparted effective antimicrobial activity against *Streptococcus mutans* (inhibition zone diameters > 10 mm) and *Porphyromonas gingivalis* (inhibition zone diameters > 25 mm). The coatings exhibited high biocompatibility, particularly at 20% and 30% concentrations, with notable cellular compatibility observed after 72 h, influenced by the roughness of the surfaces.

These findings represent a promising strategy for the fabrication of next-generation orthodontic aligners that not only restore function and esthetics but also prevent microbial complications, enhance biocompatibility with oral tissues, and potentially improve the clinical outcomes. However, further preclinical studies are necessary to assess the long-term safety, durability, and clinical applicability of this strategy.

## Figures and Tables

**Table 1:** Surface roughness test results.

Sample	(Ra, µm)
Uncoated resin disc	0.65 ± 0.25
Resin disc coated with 10% ZnO NPs	1.98 ± 0.02
Resin disc coated with 20% ZnO NPs	5.73 ± 1.08
Resin disc coated with 30% ZnO NPs	8.10 ± 0.70

## Data Availability

The authors confirm that the data supporting the findings of this study are available within the article.

## Abbreviations

The following abbreviations are used in this manuscript:

ZnO NPs	Zinc oxide nanoparticles
DMEM	Dulbecco’s Modified Eagle’s Medium
FBS	Fetal bovine serum
TSA	Trypticase soy agar
XRD	X-ray diffraction
FESEM	Field-emission scanning electron microscopy
TEM	Transmission electron microscopy
hGFs	Human gingival fibroblasts
